# Development of a Cloud-Based Image Processing Health Checkup System for Multi-Item Urine Analysis

**DOI:** 10.3390/s23187733

**Published:** 2023-09-07

**Authors:** Yu-Lin Wu, Chien-Shun Wang, Wei-Chien Weng, Yu-Cheng Lin

**Affiliations:** 1 Department of Engineering Science, National Cheng Kung University, 1 University Road, Tainan 70101, Taiwan; n98091039@gs.ncku.edu.tw (Y.-L.W.); lu4a04@gmail.com (W.-C.W.); 2 Institute of Innovative Semiconductor Manufacturing, National Sun Yat-sen University, 70 Lien-hai Road, Kaohsiung 804, Taiwan

**Keywords:** urine detection, image processing, HSV color space, color calibration, perspective transformation

## Abstract

With the busy pace of modern life, an increasing number of people are afflicted by lifestyle diseases. Going directly to the hospital for medical checks is not only time-consuming but also costly. Fortunately, the emergence of rapid tests has alleviated this burden. Accurately interpreting test results is extremely important; misinterpreting the results of rapid tests could lead to delayed medical treatment. Given that URS-10 serve as a rapid test capable of detecting 10 distinct parameters in urine samples, the results of assessing these parameters can offer insights into the subject’s physiological condition. These parameters encompass aspects such as metabolism, renal function, diabetes, urinary tract disorders, hemolytic diseases, and acid–base balance, among others. Although the operational procedure is straightforward, the variegated color changes exhibited in the outcomes of individual parameters render it challenging for lay users to deduce causal factors solely from color variations. Moreover, potential misinterpretations could arise due to visual discrepancies. In this study, we successfully developed a cloud-based health checkup system that can be used in an indoor environment. The system is used by placing a URS-10 test strip on a colorimetric board developed for this study, then using a smartphone application to take images which are uploaded to a server for cloud computing. Finally, the interpretation results are stored in the cloud and sent back to the smartphone to be checked by the user. Furthermore, to confirm whether the color calibration technology can eliminate color differences between different cameras, and also whether the colorimetric board and the urine test strips can perform color comparisons correctly in different light intensity environments, indoor environments that could simulate a specific light intensity were established for testing purposes. When comparing the experimental results to real test strips, only two groups failed to reach an identification success rate of 100%, and in both of these cases the success rate reached 95%. The experimental results confirmed that the system developed in this study was able to eliminate color differences between camera devices and could be used without special technical requirements or training.

## 1. Introduction

With the development of science and technology, more and more people in the modern world of today are experiencing so-called “diseases of civilization” [1]. However, going to a hospital for examination not only costs a lot of time and money, but also increases social costs and burdens family members. It is also difficult for people with limited mobility or with transportation problems, especially those who are elderly, to go to large medical examination centers. In addition, large-scale testing institutions usually do not inform subjects of their test results immediately. If test subjects could perform their own tests in real time, so that conditions and causes were confirmed immediately, it would be beneficial for any subsequent treatment. In 2008, Rojanathanes et al. proposed the application of gold nanotechnology to develop a rapid urine pregnancy test based on a simple process. This is one of the most widely used tests today [2]. Rapid tests have the advantage of being easy to obtain and use, and ordinary people are able to interpret their results quickly and accurately.

In this study, we used the image-capture function of smartphones to acquire images of rapid urine test strips and compare the colors obtained using a program, to assist ordinary people in interpreting their urine test results. These results were uploaded to the cloud to create a home cloud system.

### 1.1. Related Works

Urine analysis is often used by doctors when diagnosing diseases. Urine is basically composed of water and solutes. About 91–96% of urine is water [3], with solutes constituting the remainder. However, the solute composition is very complex. According to the 2011 research of Münch et al., urine solutes can mainly be divided into organic substances, hormones, proteins, drugs, tangible substances, chemical substances, and other trace elements [4]. In 2000, the advent of 10-parameter urine reagent strips (URS-10), as mentioned in Kutter’s research, greatly improved the previously cumbersome procedures associated with urine testing [5]. URS-10 are easy to operate, involve a very short period of time, and produce individually relevant test item results whose significance can then be determined.

Although URS-10 are easy to operate and involve a very short period of time, ordinary individuals without specialist training or equipment may not always be able to compare and interpret the colors of test strips with the naked eye, leading to doubts about the accuracy and completeness of any interpretation. Users may misjudge their test results, causing delays in seeking medical attention. Fortunately, due to the popularization of smartphones in recent years, almost all people now possess such a device. Compared with the human eye, it is relatively easy for machine vision to interpret URS-10 test results. In the field of machine vision, color space refers to a way of describing the numerical value of color. It is also a different way of expressing color. For example, in the printing field, the color space is converted to the CMYK color space before printing takes place [6]. Among color spaces, the most well-known one is RGB, which is widely used in displays and optical instruments [7]. In 1978, Smith proposed the concept of the HSV color space [8] which is composed of hue, saturation, and value. In 2015, Chernov et al. reported experimental results which demonstrated that HSV was more accurate than the RGB color space, and that it was close to human vision [9]. Also in 2015, Raja et al. reported that the HSV color space was more favorable than RGB for the analysis of scenes with complex light changes [10].

Color calibration is also called color restoration, and there are many ways to achieve the corrections desired when using this technique. The first color calibration card (ColorChecker) was issued by Macbeth in 1976, and it is now an indispensable form of technology in the photography, film, and television industries [11]. Using ColorChecker, correction is achieved by extracting the RGB values of 24 colors from a photographic image taken with the color calibration card. The color of the image is then restored by calculating the differences between the RGB values of the image and the reference data. In 1996, Chang et al. proposed that most RGB errors were caused by the uneven illumination of images [12]. In the present study, because images were acquired using a smartphone, it was appropriate to use the concept of a color calibration card for color calibration purposes.

Cloud computing is the model for all current computing methods. Its various applications enable many everyday tasks to be carried out more quickly and effectively, with closer relation to the specific needs of individual users. For example, in 2014, Durresi et al. proposed an architecture combining cloud computing and real-time advertising on smartphones [13]. In addition, and as mentioned in a study by Hansen et al., many large enterprises such as Microsoft and Google now regard cloud servers as an alternative form of data center which is both viable and affordable [14].

As the smartphone has become an important tool for human beings worldwide, many researchers have sought to develop detection systems based on smartphone technology in recent studies. The relevant studies include research conducted by Ra et al. in 2017 on urine analysis [15]. This study transformed subjects into the RGB color space, CIELAB color space, and HSV color space, calculating the closest distance among them to determine the detection target. In 2019, Wang et al. proposed a mobile application for urine protein detection [16], which directly extracted hue values from images for comparison. A 2022 study by Kibria et al. involved the calculation of distance by matching the RGB values of the target in images with reference RGB values [17]. All of these studies revolve around the use of smartphones for urine rapid test interpretation.

### 1.2. Contributions

The approach of this study offers users a convenient and accurate method of URS-10 interpretation, mitigating the potential medical risks resulting from misinterpretation. Besides enhancing usability, this convenient service encourages individuals to pay closer attention to their health status, subsequently reducing both medical and societal costs.

## 2. Materials and Methods

**Figure 1:**
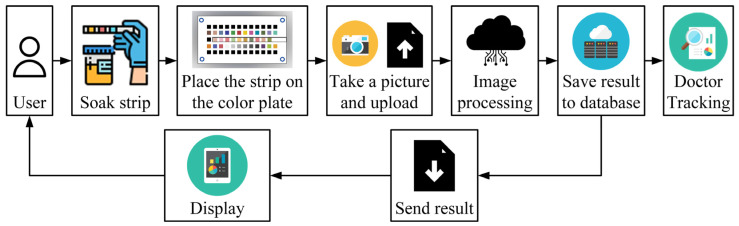
Procedure flow of the cloud-based health checkup system.

In the system used in this study, a smartphone was used to capture an image of a rapid test strip and then send the captured image to a server. The server then interpreted the test results from the image and created a user-specific folder to store both the captured image and the test results. Finally, the results of the analysis were sent back to the smartphone application and made available to the user, who could read the records in the future. The system flow is shown in Figure 1.

This system consisted of four parts: server; mobile application; hardware; and image-processing program.

### 2.1. Server

The server was managed by XAMPP, which is a free and open-source cross-platform network server management tool. In the current research, the server employed for computing the testing results was a computer host with the LINUX operating system, on which XAMPP had been installed. The functionalities of XAMPP were harnessed to establish a cloud server and a database infrastructure. The system operated by generating individualized folders within the database, delineated by user-specific IDs. These folders were designated for users to upload captured photos. Subsequently, the server undertook the task of subjecting the uploaded photos to image processing procedures.

### 2.2. Mobile Application

**Figure 2:**
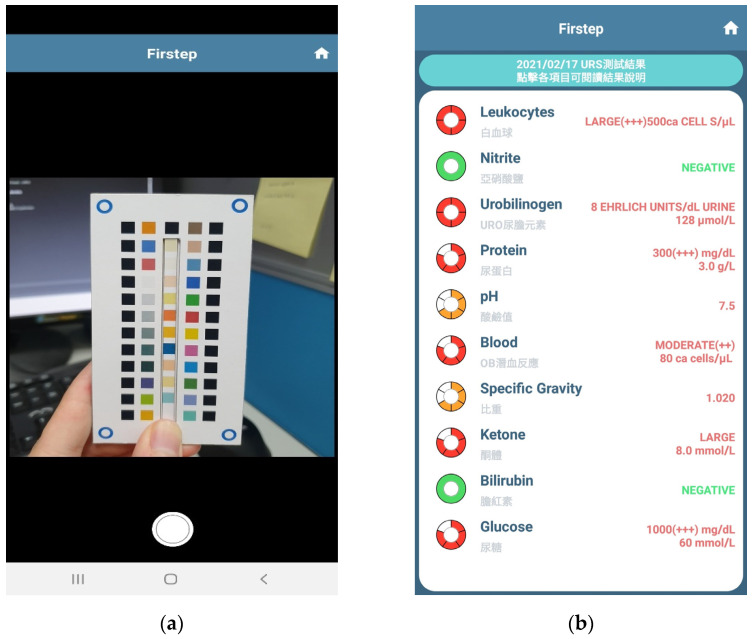
Interfaces of the mobile application: (**a**) taking a picture; (**b**) results display.

The mobile application was developed for use on both the Android and iOS systems, and it enabled users to take pictures with the built-in camera in their smartphones. The interfaces of the mobile application are shown in Figure 2. As can be seen in Figure 2a, the mobile application sent the picture to the server immediately after it was taken. If the interpretation of the image was successful, the result was then displayed on the interface, as shown in Figure 2b.

### 2.3. Hardware

**Figure 3:**
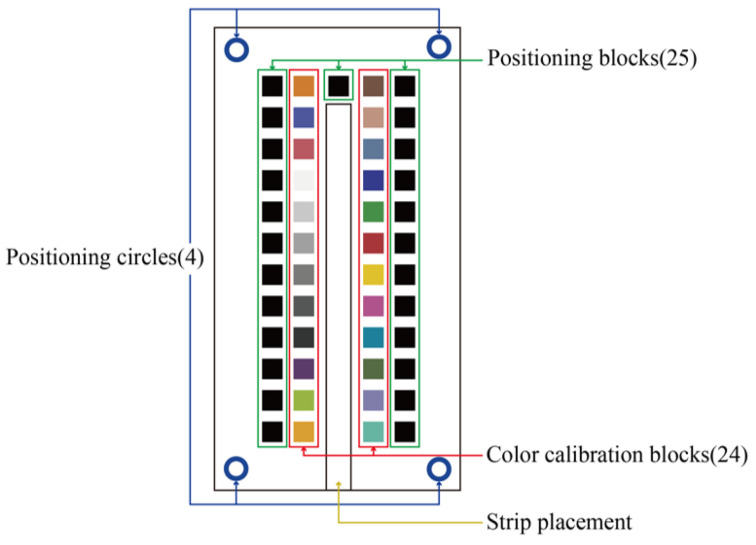
Colorimetric board used in this study.

The hardware consisted of a colorimetric board unit, with a platform designed in AutoCAD^®^ 2018 and a colorimetric board above the platform designed in Adobe^®^ Illustrator CC 2018 with a test strip placement site. Because pictures taken by users may have problems such as skew, offset, uneven sources of ambient light, motion blur, etc., the image recognition program in this study used various corrective methods, including perspective transformation, color calibration blocks, and positioning blocks, to overcome such problems. The colorimetric board designed for this study is shown in Figure 3.

### 2.4. Perspective Transformation

**Figure 4:**
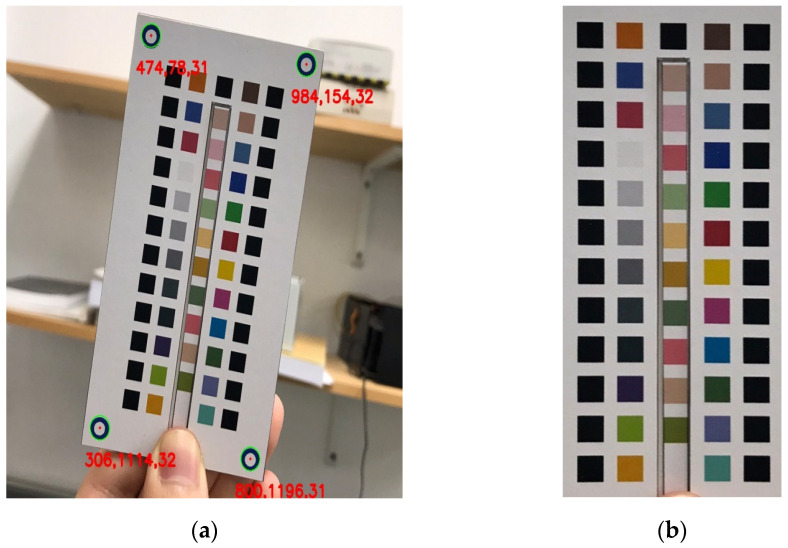
(**a**) Original image; (**b**) image after perspective transformation.

The image-processing part of the studied system served four important functions: perspective transformation; block positioning; color calibration; and color space comparison. Perspective transformation is a technique of projecting an image onto a new visual plane. It is also known as projection mapping. Perspective transformation is widely used in 3D image processing. In 2013, Do proposed a method to apply perspective transformation to process image scaling [18]. A skewed image in a 3D space can be converted to a new image plane through mapping. This process can be useful when an image is not easy to locate on the original image plane, with consequent difficulties in establishing coordinates. In the present study, we used perspective transformation to correct any angle tilt caused by the user when taking the photos. The method was to use a Hough transform to find the blue positioning circles on the colorimetric board, then use their coordinates to generate a mapping matrix, and, finally, transform the original image using the mapping matrix, thereby normalizing the image. Perspective transformations work well for de-skewing in 3D spaces. Figure 4a shows a captured image, and Figure 4b shows the image after perspective transformation.

### 2.5. Block Positioning

We used the outer black positioning color blocks as a coordinate reference to calculate the coordinates of the calibration color blocks. Because black and white values in images are very different when converted to grayscale, we regarded the place where the grayscale value changed drastically as the edge of the positioning color block, and then found the coordinates and range of the positioning blocks and calculated the positions of all the calibration color blocks. Because test strips are placed by users, the detection color blocks on the test strips may not necessarily align with the black positioning blocks; however, the same method can still be used to find the color block. In grayscale images, the position of the test strip can be found by looking for the sharp change point perpendicular to the positioning block at the top-center of the colorimetric board.

### 2.6. Color Calibration

The color calibration method involved extracting pixels from the respective RGB channels of the correction color blocks on the colorimetric board from the image after perspective transformation (as shown in Figure 4b) and then calculating the average value of the channels. The calculation equation is expressed as follows:

(1) $$X=\frac{1}{m\times n}\sum_{i=1}^{m}\sum_{j=1}^{n}X_{ij}$$

where *Xij* is the value of each channel of color block, *m* is the length of the color block, and *n* is the width of the color block. The calculated average values of the RGB channels were used as RGB values for subsequent calculations of the correction color blocks. Subsequently, 24 RGB sample data were extracted from the prestored standard sample data and expressed using the following method:

(2) $$S=\left[\begin{array}{cccc} R_{1} & R_{2} & \cdots & R_{n}\\ G_{1} & G_{2} & \cdots & G_{n}\\ B_{1} & B_{2} & \cdots & B_{n} \end{array}\right]$$

where *S* represents the aggregate of RGB values from standard samples. The values extracted from the image for the correction color block after color correction should match those of the standard samples. The average value of the RGB pixels of each color block was first transformed into a polynomial coefficient matrix *P* [19]:

*P* = [1, *r*, *g*, *b*, *r*^2^, *g*^2^, *b*^2^, *rg*, *gb*, *rb*] (3)

This represents the RGB value of a single color calibration patch after taking the image. The 24 matrices of *P* could then be used to form a new matrix *U* as follows:

(4) $$U=\left[\begin{array}{ccccc} P_{n1} & P_{nr} & P_{ng} & \cdots & P_{nrb}\\ P_{n1} & P_{nr} & P_{ng} & \cdots & P_{nrb}\\ \vdots & \vdots & \vdots & \cdots & \vdots \\ P_{n1} & P_{nr} & P_{ng} & \cdots & P_{nrb} \end{array}\right]$$

so that there was a correlation between the RGB sample matrix *S* and matrix *U*. Images could exhibit color differences due to varying shooting environments or different cameras. If color correction was applied to an image, the values of the correction color block would match those of standard samples. At this point, we could define the existence of a correction matrix that transformed *U* into *S*, and their relationship is shown as follows:

*S* = *C^T^*·*U* (5)

The color calibration matrix *C* could then be obtained through the least-squares method.

*C* = (*U*·*U^T^*)^−1^·(*U*·*S^T^*) (6)

The inner product of the uncorrected image and the color calibration matrix *C^T^* could then be used to obtain a color-corrected image.

**Figure 5:**
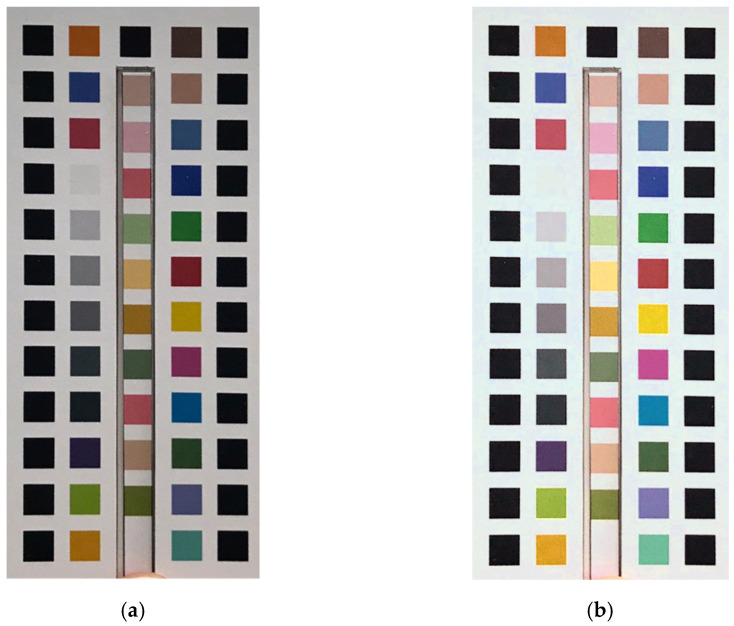
(**a**) Original image; (**b**) image after color calibration.

A comparison of pictures before and after color calibration is shown in Figure 5.

### 2.7. Color Comparison

**Figure 6:**
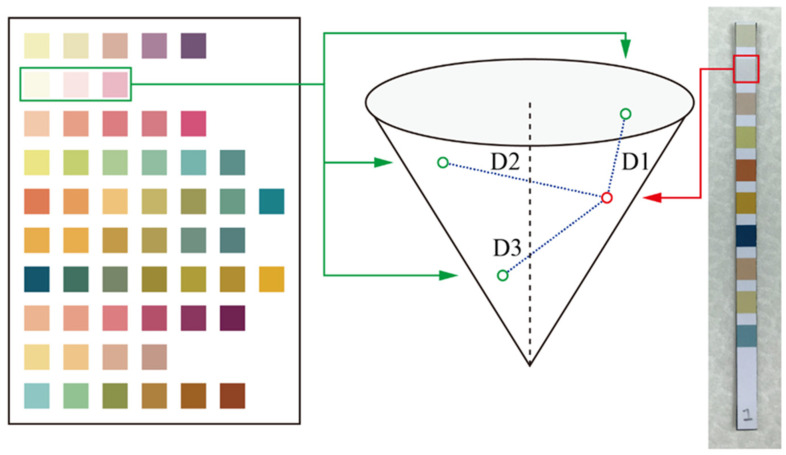
Method of color comparison used in the system.

In this study, we use the HSV color space for comparison. As mentioned earlier, HSV is suitable for environments with large changes in light sources. After color calibration, standard colors and color data from the test strips were imported into the HSV color space, and HSV color was obtained by calculating the straight-line distance between the two points used for comparison. As shown in Figure 6, the three pre-stored standard colors from the test item were imported into the HSV color space. Colors read from the test strip were then imported into the HSV color space, and the respective distances between the test sample color and the points D1, D2, and D3 in the HSV color space were then calculated.

The respective distances between the sample color and points D1, D2, and D3 could then be compared. The lesser the distance, the more similar the color. The detailed calculation method is shown in the following equation [20]:

(7) $$\mathrm{Distance}=\sqrt{{\left(V_{1}-V_{2}\right)}^{2}+S_{1}^{2}+S_{2}^{2}+S_{1}S_{2}H_{d}}$$

where *V*_1_ represents the luminance (Value) channel value of the color being tested and *S*_1_ represents the saturation channel value of the color being tested. *V*_2_ and *S*_2_ are the values of the standard sample. *H_d_* represents the difference in the hue channel between the color being tested and the standard sample. If *H*_1_ minus *H*_2_ is less than 180, *H_d_* can be represented as follows:

*H_d_* = *H*_1_ − *H*_2_ (8)

Otherwise, *H_d_* can be represented as follows:

*H_d_* = 360 − (*H*_1_ − *H*_2_) (9)

### 2.8. Standard Sample Correction

**Figure 7:**
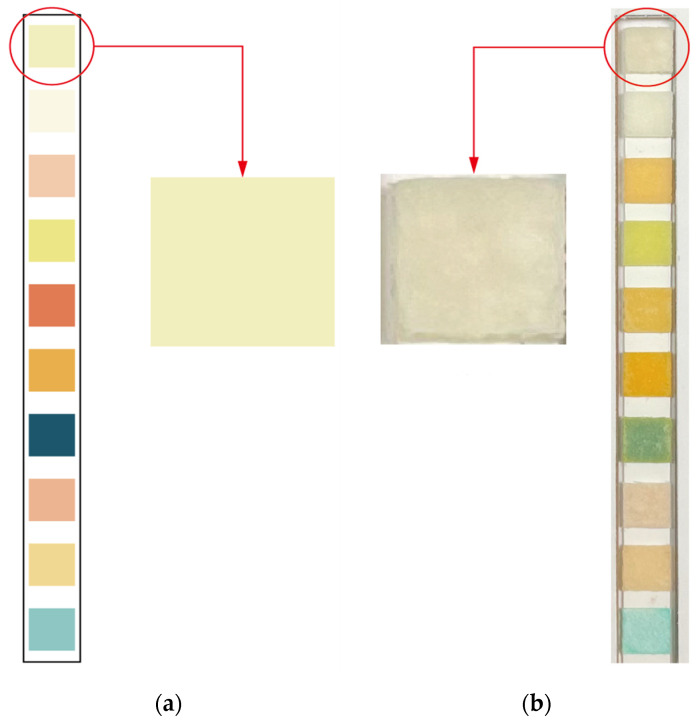
(**a**) Color image of the color chart; (**b**) color image of the real test strip.

The URS-10 test strips used in this study are a commercially available product, and the color charts provided by manufacturers may be used by inspection personnel to carry out naked-eye comparisons. The color charts were printed using general commodities, and the accuracy of the final printed color was affected by many variables, so there was a slight difference between the color of the test strip after the reaction and the printed color. Directly using the color charts supplied with commercially available products for the purpose of machine-vision recognition may have led to misjudgment, so a color chart was first used as a reference. The actual color could then be imported into the comparison chart for adjustment, so that it could be applied to image recognition, providing an accurate interpretation. As an illustration of this process, Figure 7a shows a colorimetric sample image of the color chart, and Figure 7b shows a color image of a real test strip.

Table 1 presents the HSV values of Figure 7a,b. It can be seen in the table that the HSV values of the colors of the color chart and the real test strip were different. This would have affected the calculation of distances and caused errors in the interpretation of results.

**Figure 8:**
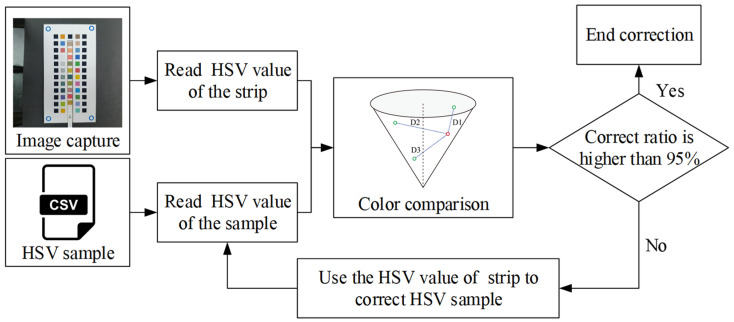
Method of HSV sample correction used in the system.

Because of this, the colors of the real test strips were used to correct HSV samples and thus improve accuracy in the interpretation of results. The correction process is shown in Figure 8.

After the correction process, the HSV values of the test strips were retrieved and compared to those of HSV samples. If the comparison accuracy was lower than 95%, the HSV samples of the color chart were corrected with the test strip until the accuracy level exceeded 95%.

### 2.9. Experimental Method

**Figure 9:**
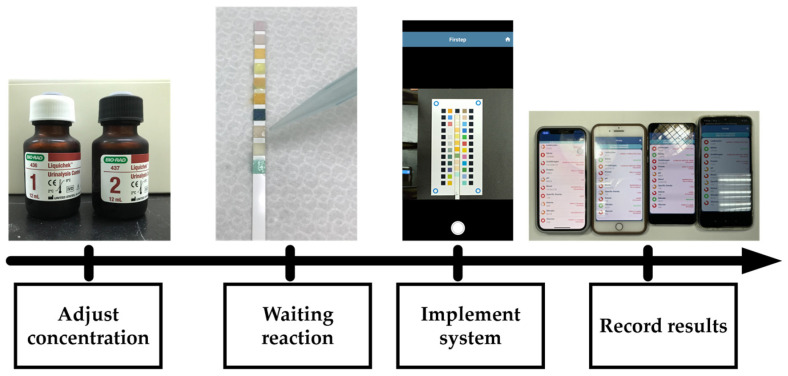
Procedure of the experimental method.

In order to verify the accuracy of the interpretation of actual test strips by our proposed system, the experiment was carried out in an indoor natural light environment by dropping urine sample specimens onto the test strips. Test sample specimens were prepared by proportionally mixing the standard urine samples Liquichek™ Urinalysis Control Level 1 and Liquichek™ Urinalysis Control Level 2 (hereafter referred to as Level 1 and Level 2). Level 1 is negative, and Level 2 is strongly positive. In this experiment, serial dilution [21] was used to prepare five concentrations of Level 2, i.e., 0%, 12.5%, 25%, 50%, and 100%. The procedure of the experimental method is shown in Figure 9.

We used four different smartphones in our experiments, namely, an iPhone 7 Plus, iPhone 12 PRO, Samsung Galaxy S10, and Redmi Note 8 Pro. Each smartphone was used 10 times for each item paired with each concentration. Due to the large amount of data generated, the data were integrated and presented as shown in following sections. Because of the limited amount of the standard sample itself, we were unable to use all possible concentrations of samples to test the test strips. Therefore, in the experiment involving actual test samples with different concentrations, each sample concentration was used only once per test strip.

## 3. Results

Experimental results prior to color correction are shown in Table 2. The 10 items listed in Table 2 are leukocytes, nitrite, urobilinogen, protein, pH, blood, specific gravity, ketone, bilirubin, and glucose. It can be seen that comparison accuracy for leukocytes, urobilinogen, and bilirubin for samples with 0% concentration was lower than that for samples with 90% concentration. For some test items, the accuracy of interpreted results was less than 50%.

The results in Table 2 indicate that the proposed method was unable to correctly identify the colors of actual sample specimens without color correction.

In order to verify the new HSV samples, two retests were carried out. The experimental method of the retest was the same as the experimental method described in the previous section. The experimental results show accuracy levels of at least 95% for each item, as shown in Table 3 and Table 4.

In the first retest, except for the pH item for samples with 12.5% concentration and the protein item for samples with 100% concentration, the accuracy rate for all items at all other concentrations was 100%. In the second retest, except for the comparison of the nitrite item for samples with 12.5% concentration, the specific gravity item for samples with 25% concentration, and the urobilinogen item for samples with 100% concentration, the accuracy rate for all items was again 100%.

The experimental results therefore reveal that comparison accuracy was improved after the color correction process.

## 4. Discussion

### 4.1. Experiments in Different Environments

Uneven light sources can be likened to introducing localized illumination during photography, causing changes in the RGB values of certain areas within the image. Such uneven light sources may be misconstrued as errors introduced by the imaging system during color correction. The ColorChecker technique employed in this study involves comparing the RGB values captured in the image with standard values and applying corrective measures. This process encompasses overall color correction of the image rather than addressing specific local regions. If the light source is uneven, it might lead to misinterpretations during conversion, resulting in an overall color distortion within the image. Example environments tested in this study, such as outdoor scenes and stairwell corners, fall within scenarios with highly uneven lighting conditions. To confirm whether our system was able to identify test strips photographed in different environments, actual test strips were used to conduct experiments in six environments with different illumination levels. The environmental data are shown in Table 5.

The experimental method was the same as in the previous experiment, and involved four different smartphones, each of which was used ten times for each item at all levels of concentration. The purpose of the experiment was mainly to explore the conditions under which recognition could correctly be achieved in various open environments. Correct numbers for all items in the same environment were counted together to obtain the accuracy rate for that environment. The experimental results are shown in Table 6.

It can be seen in these results that accuracy rates for the indoor work area and the indoor office area were relatively close. Accuracy rates for the corner of the stairs, the indoor kitchen, and the arcade were also similar. The outdoor accuracy rate was the lowest because the illumination was too high. From these results, it may be deduced that our system is sensitive to ambient light sources. Our proposed method is therefore unsuitable for outdoor environments with excessive brightness but it is suitable for indoor environments with sufficient general lighting.

### 4.2. Challenges

During the development process, numerous challenges were encountered. One of these challenges pertained to the discrepancy between the actual rapid test colors and the printed sample colors, as described earlier. Recognizing that variations in rapid test colors might also exist outside of the laboratory environment, multiple iterations were attempted before opting to employ actual rapid tests for calibration and adjustment alongside the printed sample colors.

### 4.3. Future Works

In addition to expanding the range of items under detection, future enhancements could be directed towards the image-processing segment. This might involve refining algorithms to mitigate the impact of uneven light sources, optimizing methods for image localization, and similar endeavors.

## 5. Conclusions

In this study, we successfully developed a cloud detection system applied to the URS-10 urine test. By combining software and hardware, the system was able to achieve accurate results in indoor environments. Because it was executed in the cloud, errors in image processing errors and additional delays caused by differences in devices could be reduced.

The image-processing function includes image-processing methods such as perspective transformation, color calibration, and color comparison. Through the use of these processes, the comparison accuracy of the system was improved, and factors that negatively affect detection results were eliminated as much as possible, so that the color comparison of test strips could be carried out smoothly. To confirm the performance of our system, real test strips with different sample concentrations were used for comparison purposes in a natural light environment. Initial comparison results revealed some lack of accuracy in the case of real test strips; indeed, none of the test results for the 16 groups indicated 100% accuracy, and only in five instances did accuracy reach 95%. However, after the HSV sample correction process, only two items did not reach 100% accuracy, and even these reached accuracy levels of 95%. The experimental results therefore indicate that the system was able to correctly identify test results in an indoor environment.

Even in extreme brightness ranges of 5 lux and 1464 lux, the interpretation accuracy rate was still at least 80%. Furthermore, the method proposed in this study was able to eliminate identification errors caused by a tilted placement position. When taking pictures, it was only necessary to fully capture the colorimetric board and the URS-10 test strip in the frame; alignment to the center of the frame was not required. In addition, differences between camera brands and lenses were able to be eliminated after color calibration. The method proposed in this study can be directly tested with the commercially available URS-10 product, and the system has high scalability. In the future, new detection algorithms can be added to import other rapid-test products for detection.

## Figures and Tables

**Table 1:** HSV values of Figure 7.

Image	H	S	V
(a)	55	56	242
(b)	47	43	219

**Table 2:** Accuracy of interpreted results for test strips in a natural light environment with different concentrations of samples (without color correction).

Item	0%	12.5%	25%	50%	100%
Leukocytes	85%	82.5%	100%	100%	100%
Nitrite	100%	100%	100%	100%	100%
Urobilinogen	75%	97.5%	92.5%	55%	62.5%
Protein	100%	100%	100%	100%	100%
pH	97.5%	100%	100%	75%	85%
Blood	97.5%	100%	100%	95%	100%
Specific Gravity	100%	100%	100%	100%	100%
Ketone	100%	100%	100%	100%	100%
Bilirubin	32.5%	100%	100%	100%	100%
Glucose	100%	100%	95%	80%	87.5%

**Table 3:** First retest results for test strips with different concentrations of samples in a natural light environment.

Item	0%	12.5%	25%	50%	100%
Leukocytes	100%	100%	100%	100%	100%
Nitrite	100%	100%	100%	100%	100%
Urobilinogen	100%	100%	100%	100%	95%
Protein	100%	100%	100%	100%	100%
pH	100%	97.5%	100%	100%	100%
Blood	100%	100%	100%	100%	100%
Specific Gravity	100%	100%	100%	100%	100%
Ketone	100%	100%	100%	100%	100%
Bilirubin	100%	100%	100%	100%	100%
Glucose	100%	100%	100%	100%	100%

**Table 4:** Second retest results for test strips with different concentrations of samples in a natural light environment.

Item	0%	12.5%	25%	50%	100%
Leukocytes	100%	100%	100%	100%	100%
Nitrite	100%	97.5%	100%	100%	100%
Urobilinogen	100%	100%	100%	100%	95%
Protein	100%	100%	100%	100%	100%
pH	100%	100%	100%	100%	100%
Blood	100%	100%	100%	100%	100%
Specific Gravity	100%	100%	97.5%	100%	100%
Ketone	100%	100%	100%	100%	100%
Bilirubin	100%	100%	100%	100%	100%
Glucose	100%	100%	100%	100%	100%

**Table 5:** Data for six environments with different illumination levels.

Environment	Min Illuminance	Max Illuminance
Stair corner	5 lux	11 lux
Indoor, kitchen	31 lux	52 lux
Indoor, work area	328 lux	467 lux
Indoor, office area	392 lux	518 lux
Arcade	883 lux	1464 lux
Outdoor	2460 lux	4820 lux

**Table 6:** Accuracy rates of tests in each environment.

Environment	0%	12.5%	25%	50%	100%
Stair corner	95%	92.5%	90%	90%	97.5%
Indoor, kitchen	95%	80%	92.5%	90%	97.5%
Indoor, work area	100%	97.5%	95%	100%	97.5%
Indoor, office area	100%	97.5%	100%	95%	97.5%
Arcade	90%	90%	92.5%	82.5%	97.5%
Outdoor	70%	67.5%	85%	67.5%	85%

## Data Availability

Not applicable.
